# 超高效液相色谱-串联质谱法测定尿液中7种苯系物代谢物

**DOI:** 10.3724/SP.J.1123.2022.05016

**Published:** 2023-04-08

**Authors:** Tian QIU, Xu ZHANG, Yanwei YANG, Xiaojian HU, Song LUO, Ying ZHU

**Affiliations:** 中国疾病预防控制中心环境与人群健康重点实验室, 中国疾病预防控制中心环境与健康相关产品安全所, 北京 100021; China CDC Key Laboratory of Environment and Population Health, National Institute of Environmental Health, Chinese Center for Disease Control and Prevention, Beijing 100021, China

**Keywords:** 超高效液相色谱-串联质谱, 苯系物代谢物, 生物监测, 尿液, ultra performance liquid chromatography-tandem mass spectrometry (UPLC-MS/MS), monoaromatic hydrocarbon metabolites, biomonitoring, urine

## Abstract

建立了同时测定人体尿液中7种苯系物代谢物的超高效液相色谱-串联质谱检测方法。0.5 mL尿液经盐酸水解、EVOLUTE^®^EXPRESS ABN固相萃取板(10 mg)净化、洗脱、稀释后测定。使用ACQUITY UPLC HSS T3色谱柱(100 mm×2.1 mm, 1.8 μm),以0.1%甲酸水溶液和甲醇作为流动相进行梯度洗脱,分离目标化合物,负离子电喷雾多反应监测模式下测定含量。7种目标化合物在各自范围内线性关系良好,相关系数(*r*^2^)>0.995;方法检出限为马尿酸(HA)0.9 mg/L,其余目标化合物0.02~4 μg/L,定量限为HA 3 mg/L,其余目标化合物0.05~12 μg/L;在实际尿液中低、中、高3个水平的加标回收率为84%~123%,日内精密度为1.8%~8.6%,日间精密度为1.9%~21.4%。应用该方法测定吸烟和非吸烟人群尿液样品各16份,吸烟人群中7种目标化合物检出率均为100%;非吸烟人群中反-反式黏糠酸(MU)、苄基巯基尿酸(BMA)、HA和2-甲基马尿酸(2MHA)的检出率为100%, (*S*)-苯巯基尿酸(PMA)的检出率为75%, 3-甲基马尿酸(3MHA)+4-甲基马尿酸(4MHA)的检出率为81%; MU、PMA、2MHA和3MHA+4MHA在吸烟和非吸烟人群尿液中的浓度具有统计学差异(*p*<0.001)。该方法样品用量少,可进行高通量测定,结果可靠,适用于人体尿液中7种苯系物代谢物的含量测定。

## Abstract (en)

Monoaromatic hydrocarbons (MAHs) such as benzene, toluene, and xylene are important anthropogenic pollutants in the urban atmosphere. The detection of urinary MAH metabolites are included in human biomonitoring programs in several countries, including Canada, the United States, Italy, and Germany, because their evaluation is vital to monitor the exposure of humans to MAHs. To this end, herein, a method was developed for the determination of seven MAH metabolites through ultra performance liquid chromatography-tandem mass spectrometry (UPLC-MS/MS). An aliquot of 0.5 mL urine was fortified with an isotopic labeled internal standard solution before being hydrolyzed by 40 μL of 6 mol/L HCl solution, followed by extraction using a 96-well EVOLUTE^®^EXPRESS ABN solid-phase extraction plate. The samples were washed with 1.0 mL of methanol-water (10∶90, v/v) and eluted with 1.0 mL methanol. The eluate was diluted four times with water prior to use in instrumental analysis. Chromatographic separation was achieved using an ACQUITY UPLC HSS T3 column (100 mm×2.1 mm, 1.8 μm), with gradient elution using 0.1% formic acid as mobile phase A and methanol as mobile phase B. The detection of seven analytes was performed using a triple-quadrupole mass spectrometer equipped with a negative electrospray ionization source in the multiple reaction monitoring mode. The linear ranges of the seven analytes varied from 0.1-20 μg/L to 2.5-500 mg/L, with correlation coefficients greater than 0.995. The method detection limits were 1.5, 0.02, 0.1, 900, 0.6, and 4 μg/L for *trans*,*trans*-muconic acid (MU), *S*-phenylmercapturic acid (PMA), *S*-benzylmercapturic acid (BMA), hippuric acid (HA), 2-methyl hippuric acid (2MHA), and 3-methyl hippuric acid (3MHA)+4-methyl hippuric acid (4MHA), respectively. The limits of quantification were 5, 0.05, 0.4, 3000, 2, and 12 μg/L for MU, PMA, BMA, HA, 2MHA, and 3MHA+4MHA, respectively. The method was verified by spiking urine samples at three different concentration levels, with recovery rates ranging from 84% to 123%. The intra- and inter-day precisions were 1.8%-8.6% and 1.9%-21.4%, respectively. The extraction efficiencies were 68%-99%, and the matrix effects ranged from -11% to -87%. The urine samples obtained from the German external quality assessment scheme (round 65) were used to assess the accuracy of this method. Both high and low concentrations of MU, PMA, HA, and methyl hippuric acid were within the tolerance range. All analytes in the urine samples were found to be stable for up to seven days at room temperature (20 ℃, absence of light), with less than 15% change in concentration. Analytes in urine samples were found to be stable for at least 42 d at 4 ℃ and -20 ℃, or for six freeze-thaw cycles and up to 72 h in an autosampler (8 ℃). The method was applied to the analysis of 16 non-smokers’ and 16 smokers’ urine samples. The detection rates of MU, BMA, HA, and 2MHA were 100% in both non-smokers’ and smokers’ urine samples. PMA was detected in 75% non-smokers’ and 100% smokers’ urine samples. 3MHA+4MHA was detected in 81% non-smokers’ urine and in all smokers’ urine samples. Statistical differences were found for MU, PMA, 2MHA, and 3MHA+4MHA between the two groups (*p*<0.001). The established method has good robustness and can provide reliable results. The experiments were carried out in a high-throughput manner with large sample sizes, owing to the small sample volume, and allowed the successful detection of the seven MAH metabolites in human urine.

苯、甲苯、二甲苯等苯系物被广泛用作溶剂和化工原料,常见于烟草烟雾、油漆、黏合剂和汽油中,也是室内空气挥发性有机物(VOCs)中的典型污染物^[1,2]^。苯被国际癌症研究机构(IARC)归为Ⅰ类致癌物,甲苯和二甲苯被归为Ⅲ类致癌物^[3]^,长期暴露可导致白血病或其他血液病、中枢神经系统疾病以及肝肾损伤等,严重的急性暴露亦可导致死亡^[4,5]^。常见的职业暴露环境包括木材厂、石油化工厂、皮革制品厂和加油站,室内装修和烟草烟雾则是非职业暴露人群重要的暴露来源^[6⇓-8]^。

苯系物进入人体后,经代谢,主要通过尿液排出体外。苯和甲苯的代谢主要发生在肝脏,经不同代谢途径分别生成反-反式黏糠酸(MU)和(*S*)-苯巯基尿酸(PMA),以及苄基巯基尿酸(BMA)和马尿酸(HA),并经尿液排出,其中约80%甲苯转化为HA排出体外。二甲苯在进入人体后,约95%在肝脏中代谢为甲基马尿酸(MHAs),包括2-甲基马尿酸(2MHA)、3-甲基马尿酸(3MHA)和4-甲基马尿酸(4MHA),其中70%~80% MHAs在24 h内经由尿液排出体外^[9]^。苯系物暴露的常用生物标志物包括MU、PMA、BMA、HA、2MHA、3MHA、4MHA等。其中,PMA和BMA分别是苯和甲苯暴露的特异性代谢产物。美国国家健康和营养检查调查^[10]^、加拿大人健康测量调查^[11]^、意大利生物监测^[12]^、德国环境调查项目^[13]^、美国烟草与健康评估研究^[14]^及美国职业暴露人群的日常监测^[15]^中都涵盖了以上一种或多种苯系物代谢物。

尿中苯系物代谢物常用的前处理方法包括液液萃取^[15]^、固相萃取^[11]^、直接稀释^[16]^等;尿液样品用量通常为1.0~8.0 mL^[15,17,18]^,较大的样品体积使得高通量测定难度较大,实验效率较低。检测方法包括高效液相色谱-紫外检测器(HPLC-UV)、高效液相色谱-串联质谱法(HPLC-MS/MS)、高效液相色谱-二极管阵列检测器(HPLC-DAD)、气相色谱-质谱法(GC-MS)等^[15,17⇓-19]^。其中含量水平较高(mg/L级别)的HA、MHAs可用HPLC-UV、HPLC-DAD检测,含量水平较低(μg/L级别)的PMA、BMA则通常使用HPLC-MS/MS进行测定。虽然苯系物的暴露途径相似,但鉴于各代谢物在人体内的暴露水平差异较大,鲜有方法同时测定这几种代谢物。为测得尿液中PMA总量,需要加入强酸进行水解^[20]^。现有方法通常在测定PMA总量的同时只能测定少数几种物质^[12]^,或在测定多种物质的同时测定游离状态PMA^[21]^。本方法涉及各生物标志物的化合物结构信息和在普通人群中的浓度水平见表1。本文建立了人体尿液中7种苯系物代谢物的固相萃取-超高效液相色谱-串联质谱(SPE-UPLC-MS/MS)测定方法,可在测定PMA总量的基础上,同时测定多种浓度差异较大的代谢物,具有操作简便、所需样品量少、使用溶剂量少、可高通量测定等优点。

**表1:** 7种苯系物代谢物名称、CAS编号、结构及人群中的浓度

Analyte	Formula	CAS No.	Structure	Median level	
trans, trans-Muconic acid (MU)	C_6_H_6_O_4_	3588-17-8	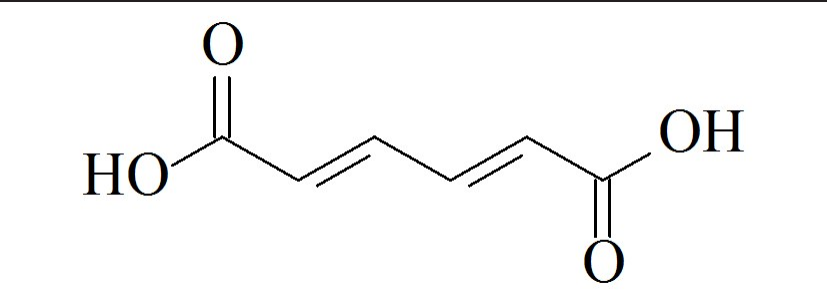	52	μg/L^[22]^
S-Phenylmercapturic acid (PMA)	C_11_H_13_NO_3_S	4775-80-8	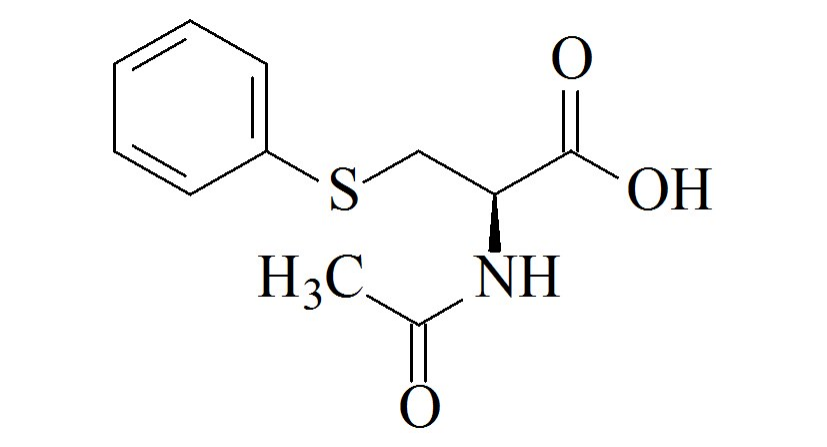	0.097	g/g crea (non-smokers),
				1.1	μg/g crea (smokers)^[12]^
				S-Benzylmercapturic acid (BMA)	C_12_H_15_NO_3_S	19542-77-9	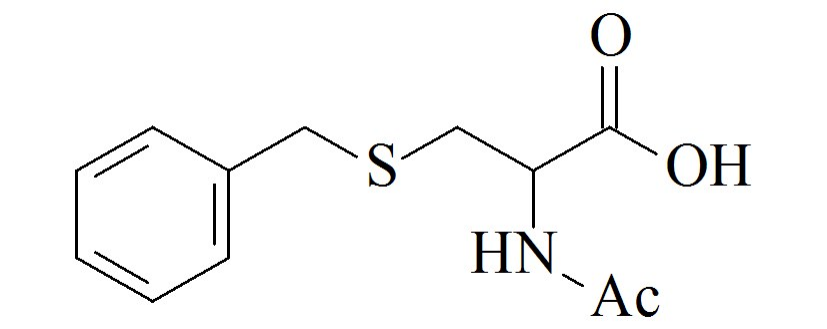	6.1	μg/L^[10]^
Hippuric acid (HA)	C_9_H_9_NO_3_	495-69-2	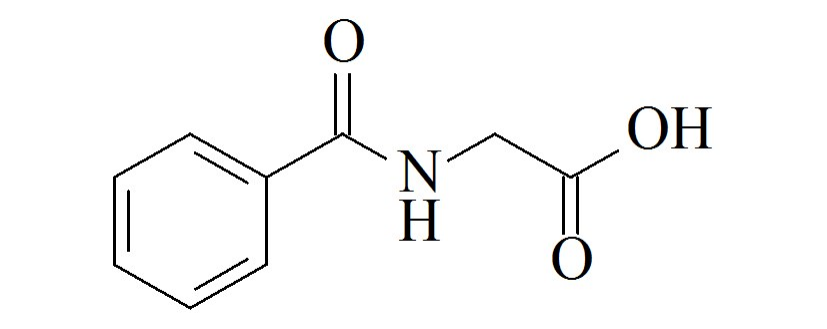	180	mg/g crea^[23]^
2-Methyl hippuric acid (2MHA)	C_10_H_11_NO_3_	42013-20-7	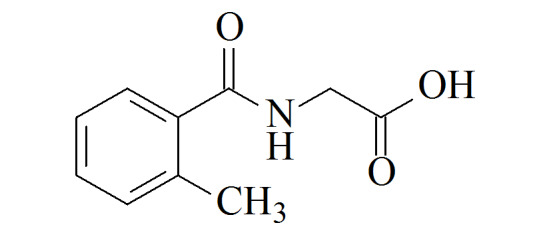	30	μg/L^[22]^
3-Methyl hippuric acid (3MHA)	C_10_H_11_NO_3_	27115-49-7	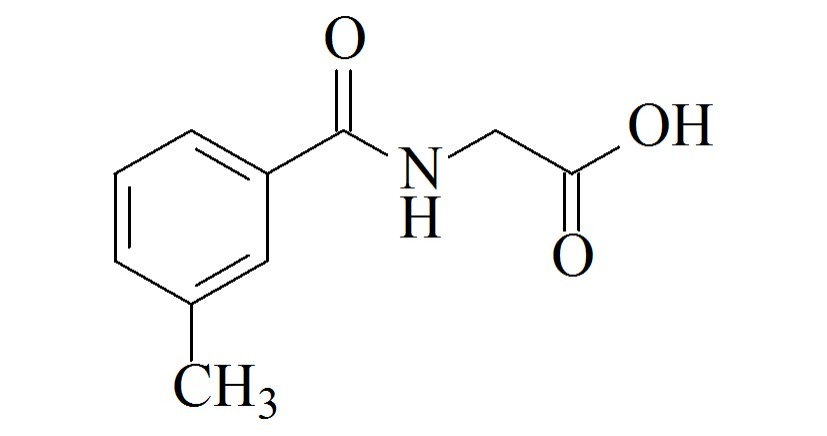	194	μg/L^[10]^
4-Methyl hippuric acid (4MHA)	C_10_H_11_NO_3_	27115-50-0	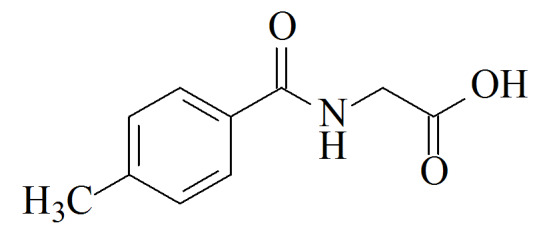		

## 1 实验部分

### 1.1 仪器、试剂与材料

Acquity I Class UPLC^®^超高效液相色谱仪(美国Waters公司), Qtrap^®^6500+三重四极杆质谱仪配电喷雾离子(ESI)源(美国AB Sciex公司); Biotage^®^Extrahera^TM^全自动样品前处理系统、96孔EVOLUTE^®^EXPRESS ABN固相萃取板(10 mg)(瑞典Biotage公司); Vortex-Genie^®^2涡旋混匀器(美国Scientific Industries公司), AP250D分析天平(美国OHAUS^®^公司)。

甲醇(色谱纯,美国Fisher公司),甲酸(HPLC级,德国CNW^®^公司), 18.2 MΩ·cm超纯水由IQ7005纯水仪(美国Millipore公司)制备,盐酸(分析纯,国药集团)。

MU、PMA、BMA、2MHA、3MHA、苄基巯基尿酸-d3(BMA-d3)、马尿酸-d5(HA-d5)、2-甲基马尿酸-d7(2MHA-d7)和3-甲基马尿酸-d7(3MHA-d7)购于加拿大TRC公司;HA购于中国J&K公司;反-反式黏糠酸-d4(MU-d4)、(*S*)-苯巯基尿酸-d2(PMA-d2)购于加拿大CDN Isotopes公司。

### 1.2 标准溶液配制

准确称取约1.0 mg PMA、BMA、2MHA、3MHA、MU-d4、PMA-d2、BMA-d3、2MHA-d7和3MHA-d7,约10.0 mg MU、HA-d5,约100.0 mg HA的标准物质,用甲醇-水(50∶50, v/v)溶解并定容至10 mL,配制成单标储备液。

分别准确移取适量各单标储备液,用甲醇-水(50∶50, v/v)稀释并定容至10 mL,配制成苯系物代谢物混合标准溶液和混合内标溶液,于-20 ℃保存待用,混合标准溶液中MU质量浓度为50.0 mg/L, PMA为1.0 mg/L, BMA、2MHA和3MHA为10.0 mg/L, HA为5000 mg/L;混合内标溶液中MU-d4质量浓度为5.0 mg/L, PMA-d2为0.1 mg/L, BMA-d3、2MHA-d7和3MHA-d7为1.0 mg/L, HA-d5为500 mg/L。

用纯水将苯系物代谢物混合标准溶液稀释至不同浓度并加入混合内标溶液,配制标准系列工作溶液。

### 1.3 实验条件与方法

#### 1.3.1 样品前处理方法

冷冻尿液在4 ℃冰箱中过夜解冻后,取出放至室温待测。充分振荡后准确移取0.50 mL待测尿液至2 mL 96孔收集板,加入40 μL 6 mol/L盐酸溶液,充分混匀后静置10 min进行水解。

依次用1.0 mL甲醇和1.0 mL纯水活化和平衡96孔EVOLUTE^®^EXPRESS ABN固相萃取板(10 mg)。使用Biotage^®^Extrahera^TM^全自动样品前处理系统向样品中加入100 μL混合内标溶液并混匀后全部转移至固相萃取板,加压使样品通过固相萃取板填料。注意压力不应过大,流速应小于1滴/s。用1.0 mL甲醇-水(10∶90, v/v)淋洗,弃去淋洗液,后用1.0 mL甲醇洗脱,收集洗脱液。移取200 μL洗脱液,加入600 μL纯水,混匀,待测。

#### 1.3.2 色谱条件

色谱柱为ACQUITY UPLC HSS T3色谱柱(100 mm×2.1 mm, 1.8 μm,美国Waters公司)。流动相A为0.1%甲酸水溶液,流动相B为0.1%甲酸甲醇溶液。梯度程序:0~3.5 min, 95%A~90%A; 3.5~8.0 min, 90%A~10%A; 8.0~9.0 min, 10%A; 9.0~9.1 min, 10%A~95%A; 9.1~11.5 min, 95%A。流速为0.3 mL/min,柱温为40 ℃,进样量为10 μL。

#### 1.3.3 质谱条件

电离模式为电喷雾负离子模式(ESI^-^),检测方式为多反应监测(MRM)模式,碰撞气(CAD)为Medium,气帘气(CUR)压力为241 kPa,雾化气(GS1)压力为345 kPa,加热气(GS2)压力为345 kPa,喷雾电压(IS)为-4000 V,离子源温度(TEM)为550 ℃。7种苯系物代谢物及其对应稳定同位素内标的保留时间、离子对、去簇电压(DP)和碰撞能量(CE)等参数见表2。

**表2:** 7种苯系物代谢物及其稳定同位素内标的保留时间和质谱参数

Analyte	Retention time/min	Ion pairs (m/z)	Declustering potentials/V	Collision energies/eV
MU	4.7	141>53^*^, 141>97, 141>59	-20, -20, -20	-16, -12, -15
PMA	7.7	238>109^*^, 239>110	-10, -20	-13, -13
BMA	7.8	252>123^*^, 253>124	-20, -20	-17, -22
HA	6.6	178>160^*^, 178>133	-20, -20	-10, -20
2MHA	7.0	192>91^*^, 192>148, 192>174	-20, -20, -20	-20, -15, -10
3MHA & 4MHA	7.4	192>91^*^, 192>148, 192>174	-20, -20, -20	-15, -15, -10
MU-d4	4.7	145>100	-10	-11
PMA-d2	7.7	240>109	-20	-27
BMA-d3	7.8	255>123	-20	-20
HA-d5	6.6	183>139	-20	-17
2MHA-d7	7.0	199>155	-65	-16
3MHA-d7 & 4MHA-d7	7.3	199>155	-20	-16

3MHA and 4MHA had identical parameters, same with 3MHA-d7 and 4MHA-d7. * Quantitation ion.

## 2 结果与讨论

### 2.1 前处理条件优化

#### 2.1.1 酸的选择

尿液中存在一定量的PMA前体(pre-PMA),是谷胱甘肽代谢路径上的代谢产物^[24]^,在酸性条件下可水解生成PMA^[25]^。尿液中pre-PMA占PMA总量的比例因人而异,研究显示在职业暴露人群和吸烟人群中,尿液中pre-PMA可占到PMA总量的75%~99%^[25,26]^。前处理过程中若未经酸水解,只能测得游离状态的PMA;只有经过酸处理才可测得PMA的总暴露量,更能客观反映真实的暴露水平。

本实验比较了甲酸、盐酸、硫酸等不同种类酸及其用量对尿中PMA含量测定结果的影响。取1.0 mL吸烟人群尿液样品,分别加入10~50 μL甲酸、浓盐酸或浓硫酸,充分振荡、水解10 min后进行前处理和测定。结果如图1所示,PMA测定结果出现平台期,即酸用量大于40 μL时,PMA测定结果不再上升,说明水解反应已达到平衡。其中,使用甲酸时测得的PMA含量低于使用盐酸或硫酸时的含量,可能的原因是甲酸属于弱酸,高pH环境不足以使结合态的PMA完全水解,而盐酸和硫酸属于强酸,低pH值足以完全水解结合态PMA。但考虑到浓硫酸氧化性强,为避免尿液中的其他物质氧化干扰目标化合物的测定,最终选择使用盐酸进行酸解。实验还比较了在加入40 μL浓盐酸3、5、10或30 min后加入40 μL 50%氢氧化钠溶液终止反应后进行前处理和测定的结果。结果显示,PMA浓度没有因反应时间长短而发生明显变化,以上4种条件测得PMA浓度偏差小于5%。因浓盐酸挥发性强、液体黏度低、小体积移取时误差较大,故测定实际样品时增大移取液体的体积(每500 μL尿液加入40 μL 6 mol/L盐酸溶液),方便实验操作,提高实验精度。

**图1:**
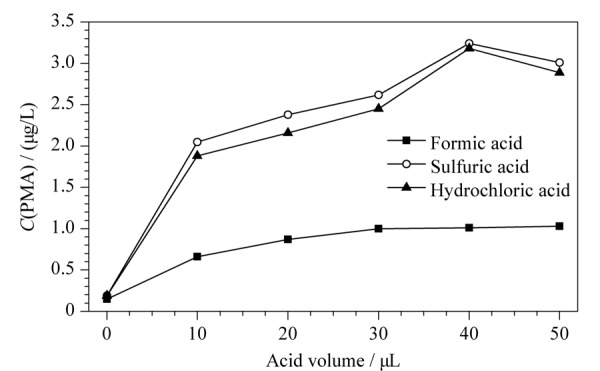
酸种类及用量对尿中PMA含量的影响

#### 2.1.2 样品净化条件的优化

在尿液中加入内标,使用固相萃取小柱对样品进行前处理。分别使用甲醇以及不同比例的甲醇水溶液(10%~90%,以10%为梯度)1.0 mL进行淋洗,并收集淋洗液进行上机测定。结果显示,使用甲醇-水(10∶90, v/v)淋洗时,淋洗液中未检出各内标;使用甲醇-水(20∶80, v/v)淋洗时,淋洗液中可检出MU-d4,提示甲醇-水(20∶80, v/v)可将样品中的MU洗脱下来,造成前处理净化过程中的损失。因此,选择甲醇-水(10∶90, v/v)淋洗净化样品,以完整保留目标化合物。

氮吹浓缩是富集低浓度目标化合物的常用前处理方式,然而本实验发现,PMA的基质抑制效应非常严重,浓缩不仅不能有效提高目标化合物的质谱响应,反而会影响方法检出限,适度的稀释则是降低基质效应简单易行的方法。实验比较了将1.0 mL洗脱液氮气吹干后复溶于100 μL纯水(浓缩10倍)、将1.0 mL洗脱液氮气吹干后复溶于1.0 mL纯水(不浓缩也不稀释)、取200 μL洗脱液加入600 μL纯水(稀释4倍)、取100 μL洗脱液加入900 μL纯水(稀释10倍)等不同处理方式,发现与稀释4倍洗脱液相比,浓缩10倍与不浓缩也不稀释两种条件下,PMA的目标物峰面积并没有显著提高,但稀释10倍洗脱液时,PMA的目标物峰面积有明显的降低。最终确定稀释4倍的处理方式可以兼顾降低基质效应和维持较低的方法检出限。

#### 2.1.3 色谱条件的选择

分别比较了5 mmol/L酸性甲酸铵溶液(pH 3)、0.1%甲酸水溶液(pH 2.7)、纯水、6.5 mmol/L乙酸铵溶液(pH 6.5)作为流动相A, 0.1%甲酸甲醇溶液、纯甲醇作为流动相B的情况下,各目标化合物的分离效果和质谱响应强度。结果显示,使用0.1%甲酸水溶液为A相时,各目标化合物的响应高于使用其他几种流动相;而流动相B的种类对结果影响不大。最终选择0.1%甲酸水溶液为A相、甲醇为B相,此条件下各目标化合物峰形对称,峰宽窄,信号强度高,分离度良好(见图2)。

**图2:**
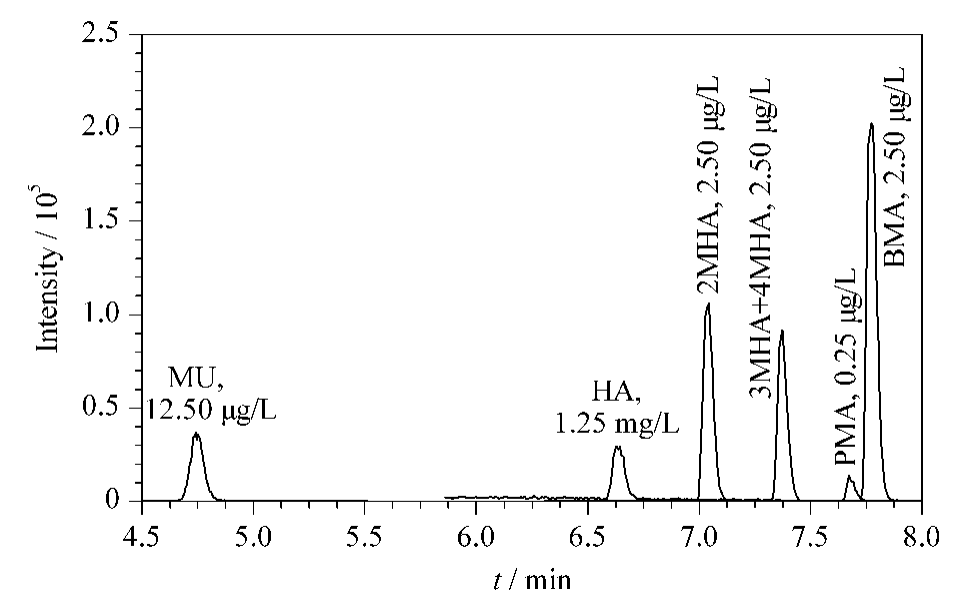
7种苯系物代谢物的总离子流色谱图

比较了Waters ACQUITY HSS T3色谱柱和Waters ACQUITY BEH C_18_色谱柱,前者的疏水性弱于后者,更适宜保留和分离极性强的物质。以HA-d5为例,使用C_18_柱无法较好地分离目标峰和干扰峰,而使用T3柱则可以分开,保证积分和定量的准确(见图3)。

**图3:**
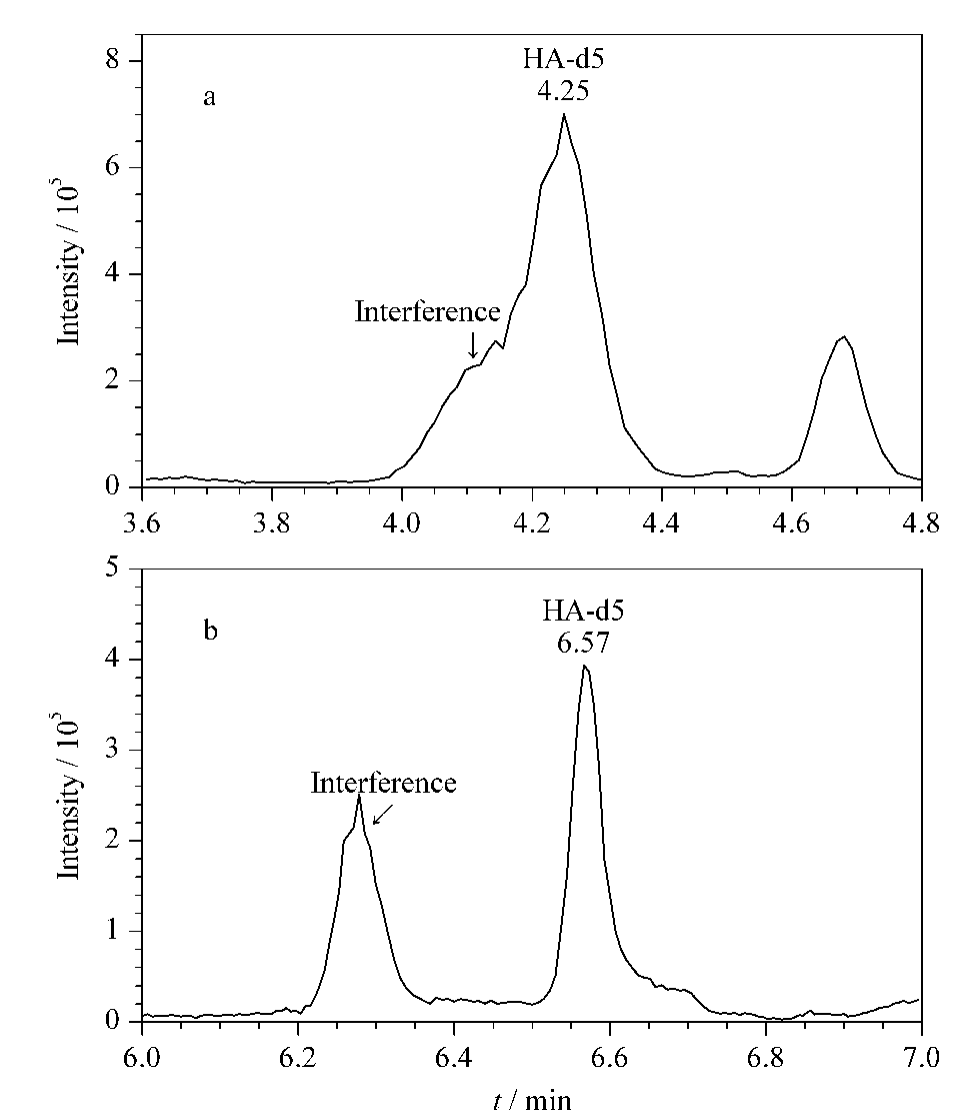
在使用(a)ACQUITY BEH C_18_和(b)ACQUITY HSS T3色谱柱时HA-d5的选择离子色谱图

#### 2.1.4 质谱条件的选择

与其他同类方法相比,本方法并非所有目标化合物都选用了仪器响应最高的离子对作为定量离子对。以MU为例,相比141>53离子对,141>97离子对的离子流色谱图具有更高的信号响应,但其基线也更高,在同等浓度下,信噪比低于141>53离子对(见图4)。此外,在测定实际尿液时,141>97离子对色谱图中目标化合物出峰受到基质干扰的情况更加常见,而141>53离子对的色谱图中,除目标化合物峰外,鲜有干扰峰出现,故最终选择离子对141>53作为定量离子对,离子对141>97作为定性离子对,并优化离子对141>59作为第二对定性离子对,以便在定性离子受到基质干扰时对目标化合物依据离子丰度比进行定性。基于同样的原因,MHAs选择离子对192>91作为定量离子对,离子对192>148作为定性离子对,并优化离子对192>174为第二对定性离子对。

**图4:**
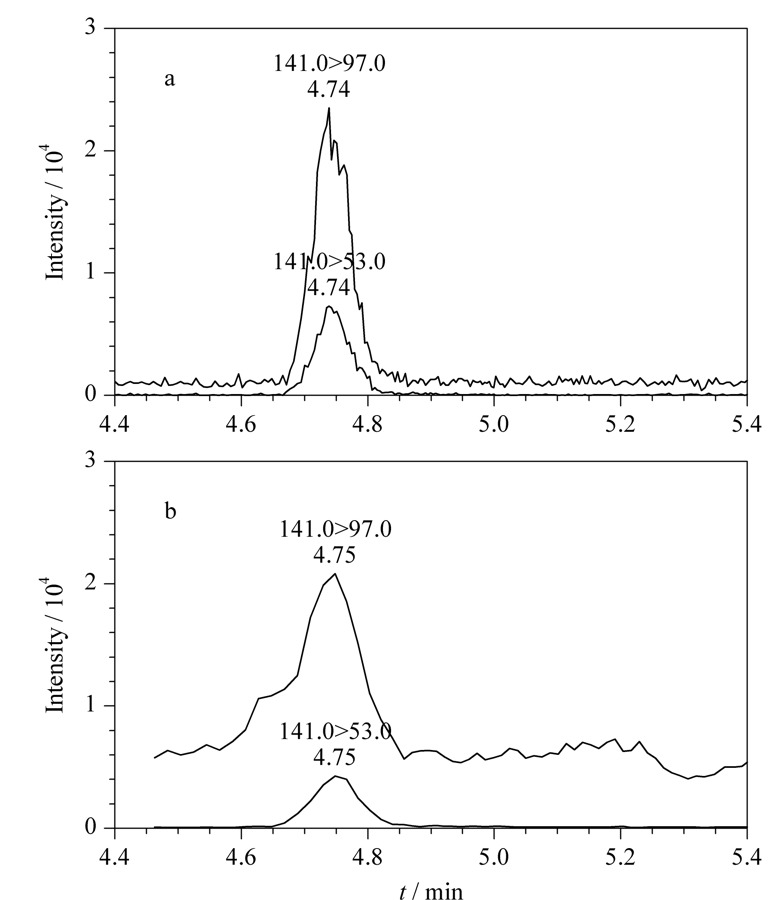
MU在(a)溶剂和(b)实际尿液中定量离子对(141>53)和定性离子对(141>97)的选择离子色谱图

因HA在尿液中含量较高,如果使用仪器响应最高的离子对(178>134)进行定量,则很容易出现检测器饱和的情况。因此在优化离子对时,选择了仪器响应较低的离子对(178>160和178>133)进行定量和定性,以达到可以与其他含量水平较低的目标化合物同时测定的目的。

PMA常见的稳定同位素内标包括PMA-d2、PMA-d5和PMA-^13^C_6_ 3种,在实际样品测定过程中发现,PMA-d5和PMA-^13^C_6_容易有基质干扰,影响内标峰的积分,而PMA-d2出峰前后鲜有干扰峰,方便准确定量(见图5)。此外,虽然PMA-d2与PMA定量离子对选择的子离子是相同的,但我们使用PMA-d2单标溶液单独进样(浓度与样品测定时加入内标浓度相同),未发现在目标化合物质谱通道出峰(见图6),排除了检测通道交叉污染的潜在影响。综合以上因素,选择使用PMA-d2作为内标。基于同样的原因,选择BMA-d3而非BMA-d5作为BMA的稳定同位素内标。

**图5:**
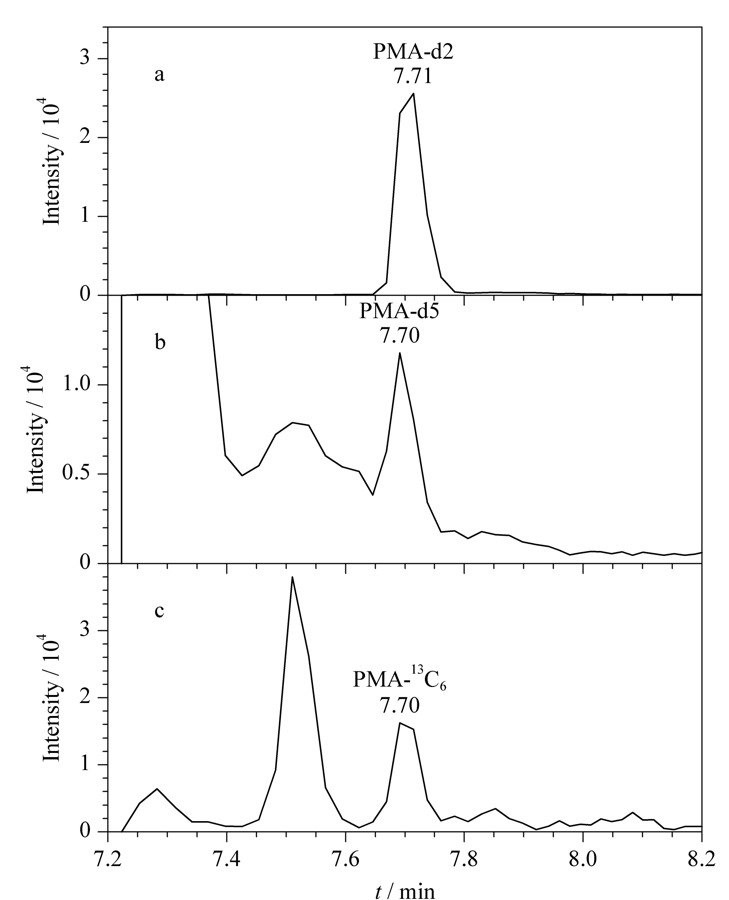
(a)PMA-d2、(b)PMA-d5和(c)PMA-^13^C_6_在尿液中的选择离子色谱图

**图6:**
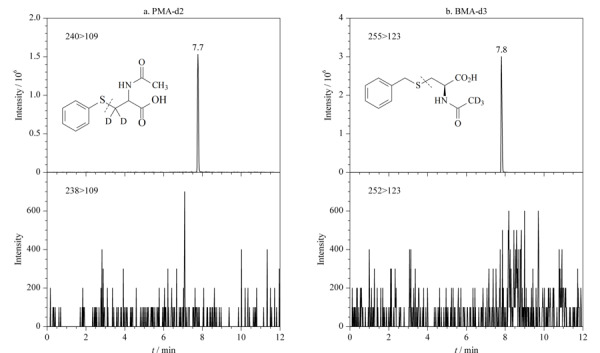
(a)PMA-d2、(b)BMA-d3的选择离子色谱图

### 2.2 前处理萃取效率

取不同尿液样品,每个样品分为2组,第一组在前处理前加入高、中、低3个不同水平的内标溶液,并进行前处理;第二组样品先进行前处理,然后加入不同水平内标溶液,上机测定。以前处理前、后加入内标样品的信号响应峰面积的比值计算前处理萃取效率。结果显示,7种苯系物代谢物的前处理萃取效率为68%~99%。

### 2.3 基质效应评价

基质效应参照Panuwet等^[27]^的方法进行评估,即取不同尿液样品,经前处理后收集洗脱液,并在洗脱液中加入内标,上机测定;另在水中加入等量内标进行上机测定。以(洗脱液中内标峰面积-溶剂中内标峰面积)/溶剂中内标峰面积,计算基质效应。基质效应<0为抑制作用,>0则为增强作用。经计算,7种苯系物代谢物的基质效应为-11%~-87%。总体而言,尿液对各目标化合物测定的抑制作用较为明显,且由于每个尿液样品基质都有差异,在实际样品测定时无法预料具体样品的基质效应强弱,个别样品过强的抑制作用可能会影响方法灵敏度,美国CDC实验室数据显示约5%样品因基质效应严重影响定量^[10]^。通过加入与目标化合物一一对应的内标,可以在一定程度上校正尿液基质带来的抑制作用。

### 2.4 方法学考察

#### 2.4.1 方法检出限和定量限

配制系列标准溶液,并上机测定,以样品中目标物质量浓度为横坐标,目标物与其对应内标峰面积之比为纵坐标,绘制标准曲线。各物质在其各自范围内线性关系良好(相关系数(*r*^2^)均大于0.9995)。

参照美国环保署(US EPA)方法检出限测定程序文件(第二版)^[28]^,计算方法检出限和定量限。具体流程如下:1)本方法各目标化合物没有空白干扰,故使用标准曲线低浓度点估计各目标化合物在3~5倍信噪比时对应的浓度,作为预估的检出限;2)选择2~10倍预估检出限浓度作为加标浓度;3)准备3批次样品,含空白样品和空白加标样品,经前处理后进行上机测定,上机须分别在3个自然日进行;4)分别计算各目标化合物在空白样品和空白加标样品测定浓度的标准偏差(SD); 5)以3倍SD计算方法检出限,以10倍SD计算定量限。相比直接使用仪器3倍和10倍信噪比作为方法检出限和定量限,本方法将前处理过程的损失纳入了方法检出限的计算,因此更加接近实际样品测定时的检测能力(结果见表3)。经过计算,各目标物质的方法检出限为0.02~900 μg/L;定量限为0.05~3000 μg/L。

**表3:** 7种苯系物代谢物的回归方程、相关系数、线性范围、方法检出限和定量限

Analyte	Regression equation	r^2^	Calibration range/(μg/L)		MDL/(μg/L)	LOQ/(μg/L)	C(IS)/(μg/L)
MU	Y=5.8×10^-2^X+4.5×10^-2^	0.998	5-	1000	1.5	5	125
PMA	Y=5.2×10^-2^X+6.0×10^-4^	0.998	0.1-	20	0.02	0.05	2.5
BMA	Y=2.7×10^-2^X+6.6×10^-3^	0.999	1-	200	0.1	0.4	25
HA	Y=6.7×10^-4^X+3.2×10^-4^	0.998	2500-	500000	900	3000	12500
2MHA	Y=4.4×10^-2^X+4.8×10^-3^	0.999	1-	200	0.6	2	25
3MHA+4MHA	Y=3.7×10^-2^X+2.6×10^-2^	0.995	5-	1000	4	12	25

*Y*: peak area ratio of the quantitative ion of the analyte to the internal standard; *X*: mass concentration, μg/L.

#### 2.4.2 回收率和精密度

以实际尿液进行7种苯系物代谢物低、中、高3个水平的加标试验,每个水平进行6次平行试验,计算日内精密度和回收率;在不同自然日(*n*=3)进行上述试验,计算日间精密度。如表4所示,各目标化合物在低、中、高3个水平下的加标回收率为84%~123%,日内精密度为1.8%~8.6%,日间精密度为1.9%~21.4%。

**表4:** 尿液中7种苯系物代谢物在3个加标水平下的回收率和日内、日间精密度(*n*=6)

Compound	Spiked/ (μg/L)	Recovery/ %	Intra-day precision/%	Inter-day precision/%
MU	10.0	113	3.5	7.1
	100	106	2.0	5.7
	1000	104	3.9	4.5
PMA	0.2	105	4.9	7.9
	2.0	101	6.3	3.3
	20	108	8.6	1.9
BMA	2.0	104	2.8	7.6
	20.0	103	1.8	3.0
	200	105	3.2	3.5
HA	1000	86	5.0	21.4
	10000	84	3.6	14.5
	100000	85	2.8	6.1
2MHA	2.0	111	2.9	4.1
	20.0	103	3.3	3.1
	200	106	4.7	3.2
3MHA+4MHA	2.0	109	8.2	4.1
	20.0	123	4.2	5.4
	200	119	7.3	4.7

#### 2.4.3 稳定性试验

将尿液样品采集分装后,分别储存于室温(20 ℃,避光)、4 ℃和-20 ℃条件下,并于第0、1、3、7、14、21、42天取出进行测定。结果显示,尿液样品可在室温条件下保存7天,在4 ℃和-20 ℃条件下可保存42天。在室温(20 ℃,避光)条件下,至第7天时,样品中除2MHA外各目标化合物含量与第0天相比偏差均小于10%, 2MHA含量偏差小于15%;至第14天时,BMA、HA和3MHA+4MHA的含量均降低至方法检出限附近或检出限以下;储存于4 ℃和-20 ℃的样品中除2MHA外各目标化合物含量至第42天时的偏差均小于15%, 2MHA在样品中的含量呈缓慢降低趋势,偏差小于20%。样品采集分装后,储存于-20 ℃条件下,反复冻融并进行测定。结果显示冻融6次的尿液样品中各目标化合物含量没有明显变化(除2MHA外各物质含量变化均小于15%, 2MHA含量变化小于20%)。

样品经前处理净化后存放于进样器(8 ℃)中,在第0、4、8、16、24、48、72 h分别测定目标化合物含量。结果显示,在实验时间范围内,各目标化合物测定含量的变化均小于15%。

以上结果与其他文献基本一致。Alwis等^[16]^发现尿液样本在4 ℃、-20 ℃条件下可稳定储存一周,10个冻融周期保持稳定。美国职业卫生的方法显示待测样液在自动进样器(8 ℃)条件下可稳定6天,在室温避光条件下可稳定6天,在室温见光条件下不稳定^[29]^。Schwedler等^[13]^发现尿液样本在室温可稳定储存24 h,待测样液在自动进样器(10 ℃)中可稳定24 h,在6个冻融周期保持稳定。

#### 2.4.4 正确度评估

使用本方法对第65届德国外部质量评估项目G-EQUAS(65)样品(代码3A、3B、17A和17B)进行测定,结果见表5。该项目指标并未区分2MHA、3MHA和4MHA,因此以3种目标化合物质量浓度之和作为MHAs含量与参考值和参考范围进行比较。结果显示,MU、PMA、HA和MHAs在高、低两个水平的样品中,测定值均在参考范围之内,表明本方法正确度良好。

**表5:** 第65届德国外部质量评估项目样品中苯系物代谢物的测定结果

Analyte	Sample code	Content/(μg/L)	Reference value/(μg/L)	Tolerance range/(μg/L)	Difference/%
MU	3A	210	220	160-280	-4.5
	3B	1740	1460	1100-1820	19
PMA	17A	1.9	2.1	1.5-2.7	-9.5
	17B	24.0	23.7	19.2-28.2	1.3
HA	3A	574000	585000	507000-663000	-1.9
	3B	1233000	1389000	1227000-1551000	-11
MHAs	3A	238	224	181-267	6.4
	3B	1328	1394	1188-1600	-4.7

### 2.5 实际样品测定

使用本方法对32份实际尿液样品进行测定,其中吸烟人群16份,非吸烟人群16份,结果见表6。7种目标化合物在吸烟人群中的检出率均为100%;在非吸烟人群中,MU、BMA、HA和2MHA的检出率为100%, PMA的检出率为75%, 3MHA+4MHA的检出率为81%。2种苯代谢物(MU和PMA)以及3种二甲苯代谢物(2MHA和3MHA+4MHA)在吸烟和非吸烟人群尿液中的浓度具有统计学差异(*p*<0.001),提示烟草烟雾暴露是这4类代谢物的重要来源。该结果与现有文献^[11⇓-13,16]^结果基本一致。

**表6:** 7种苯系物代谢物在吸烟和非吸烟人群尿液中的测定值

Analyte	Median (P_25_, P_75_)/(μg/L)				p value
	Non-smokers (n=16)		Smokers (n=16)		
MU	18.4	(12.5, 29.0)	40.9	(31.8, 108)	<0.001
PMA	0.15	(0.06, 0.25)	1.06	(0.43, 2.20)	<0.001
BMA	6.33	(2.53, 14.3)	13.17	(3.97, 16.7)	0.423
HA	148000	(49400, 240000)	102000	(66200, 708000)	0.780
2MHA	15.1	(10.5, 23.0)	46.2	(24.6, 99.8)	<0.001
3MHA+4MHA	51.2	(21.5, 69.0)	202	(81.4, 551)	<0.001

P_25_: 25^th^ percentile; P_75_: 75^th^ percentile.

## 3 结论

本文建立了超高效液相色谱-串联质谱测定人体尿液中7种苯系物代谢物的分析方法,对前处理条件和仪器方法进行了优化,评估了方法学指标、基质效应和稳定性,并应用于实际样品的测定。本方法操作简便,样品使用量少,可实现96孔板高通量测定,提高检测效率;定量准确,检出限低,可满足生物样本检测,为开展人体生物检测项目提供良好的实验室技术支持。
